# Leisure and health benefits among Korean adolescents with visual impairments

**DOI:** 10.1080/17482631.2018.1435097

**Published:** 2018-03-07

**Authors:** Junhyoung Kim, Se-Hyuk Park

**Affiliations:** a Department of Health and Human Performance, Texas State University, San Marcos, TX, USA; b Department of Sports Sciences, Seoul National University of Science and Technology, Seoul, South Korea

**Keywords:** Leisure, leisure skills, perseverance, personal growth, psychological benefits, self-expression, visual impairment

## Abstract

**Purpose:** The purpose of the study was to explore health benefits through leisure engagement among Korean adolescents with visual impairments. **Method:** Using semi-structured interviews, a total of 14 adolescents with visual impairments participated in this study. **Results:** Two salient themes were captured as health benefits as a result of leisure engagement: psychological wellbeing and personal growth. **Conclusions:** The findings suggest that leisure provides a venue for the development of self-expression, leisure skills, perseverance, and positive affects. It also indicates that leisure can serve as a vehicle for promoting health and life satisfaction among Korean adolescents with visual impairments.

## Introduction

Visual impairment refers to a sight limitation that interferes with interaction with the physical environment and entails special accommodation and care to acquire information. Visual impairment challenges individuals’ abilities to participate in daily activities and interact effectively with the physical environment. Prior studies have suggested that individuals who have visual impairments tend to be dependent upon others, have decreased capabilities to perform daily activities, and experience negative psychological symptoms, such as isolation, depression, and anxiety (Burmedi, Becker, Heyl, Wahl, & Himmelsbach, 2002; Chou, 2008; Keeffe, 2005). Such challenges generate psychological distress and stress that diminish quality of life (Chou, 2008).

Leisure scholars have suggested that individuals with disabilities and/or illnesses use leisure as a way of fostering social inclusion and coping with stress (Kim, Kim, Heo, & Lee, 2015; Patterson & Pegg, 2009). By participating in various types of leisure activities, individuals with disabilities and/or illnesses mastered their leisure skills, attained positive self-image, and improved their self-expression skills (Peterson et al., 2008; Reynolds, 1997; Rubin, 2005). Such an advancement of leisure skills and self-expression contributes to quality of life and life satisfaction.

A few empirical studies have stressed the importance of leisure engagement for health benefits among individuals who have visual impairments. Park, Chong, and Kim (2015) found that individuals who have visual impairments used music as a means of expressing their feelings and emotions. They also suggested that participants developed the ability to deal with stress through music programs. In addition, Jessup, Cornell, and Bundy (2010) provided evidence that individuals who have visual impairments increased their sense of belonging and connectedness through leisure engagement. Such feelings of social inclusion helped them gain resilience (Jessup et al., 2010).

In spite of the known benefits of leisure among individuals who have visual impairment, the majority of prior studies have mainly focused on leisure and health among Westerners who have visual impairments (Cavet, 1998; Gronmo & Augestad, 2000; Rosenblum, 2000). In addition, there is a paucity of literature that explores health benefits through leisure among adolescents who have visual impairments. For this study, we selected Korean adolescents who have visual impairments as a sample group for a couple of reasons. First, even though the number of individuals in Korea who have visual impairments has increased drastically every year (Ministry of Health & Welfare in Korea, 2014), little attention has been paid to this group by researchers. From a cultural perspective, Koreans tend to have negative attitudes toward individuals with a disability and perceive having disabilities as a token of disgrace to a family (Kyun, 2000). With negative public perceptions on a disability, disability studies have been underdeveloped. Second, Korean adolescents who have visual impairments have reported experiencing higher levels of stress and more negative psychological symptoms than their peers who do not have visual impairments (Kim, 2004). As such, it is important to understand how adolescents who have visual impairments can gain health benefits through leisure participation.

Therefore, based on gaps in the current literature, the purpose of this study was to explore health benefits through leisure engagement among Korean adolescents who have visual impairments. Three questions were used to guide this study:

1. What do Korean adolescents who have visual impairments do for fun or entertainment in their free time (i.e., leisure activities)?
2. Why do Korean adolescents who have visual impairments participate in these activities?
3. What benefits do Korean adolescents who have visual impairments get from leisure participation?

### Experiences of Korean adolescents who have visual impairments

Visual impairments are considered to be one of the most debilitating and disabling conditions (Kim, 2003). As such, individuals who have visual impairments are likely to perceive psychological distress, fear, anxiety, and social isolation (Kim, 2003). Prior studies have shown that Korean adolescents who have visual impairments experienced high levels of stress due to social and peer rejection in their schools and communities (Gwon, Kim, & Lee, 2011; Lee & Oh, 2011; Lee, Park, & Kwak, 2003). Additionally, Korean adolescents who have visual impairments experience some difficulties in adjusting to their school and performing self-regulated learning abilities and academic self-efficacy (Lee & Oh, 2011; Lee et al., 2003).

Compared to peers, Korean adolescents who have visual impairments reported experiencing more negative psychological symptoms associated with their disabling conditions (Kim, 2003; Lee & Seo, 2002). For example, they experienced higher levels of depression, anxiety, and hopelessness and exhibited more deficiency in social skills and lower self-esteem than their non-visually impaired peers (Kim, 2003; Lee & Seo, 2002). As such, it appears visual impairments can lead to a low quality of life and life satisfaction for Korean adolescents.

### Health benefits through leisure engagement among individuals with disabilities

Empirical studies have demonstrated that participation in leisure promotes a perception of health and life satisfaction among individuals with disabilities (Chun & Lee, 2010; Kim et al., 2015). They provided evidence that leisure helped individuals with disabilities to develop coping strategies, experience personal growth, and improve an acceptance of their disabilities. For example, Yampolsky, Wittich, Webb, and Overbury (2008) examined the relationship between spiritual activities and coping behaviors among visually impaired individuals. They found that the individuals who were actively engaged in spiritual activities demonstrated positive adaptive coping behaviors.

Leisure produces a context in which individuals who have visual impairments can facilitate healthy adaptations to their conditions and gain physical and social benefits. Leisure scholars found that leisure engagement helped individuals who have visual impairments to pursue active leisure participation and increase their leisure motivation (Kim & Kim, 2008, 2013; Södergren, Sundquist, Johansson, & Sundquist, 2008). For example, in a school setting, visually impaired adolescents who regularly participate in leisure activities reported that they improved self-esteem and confidence (Cavet, 1998; Gronmo & Augestad, 2000; Rosenblum, 2000).

A few researchers have explored ways in which Korean adolescents who have visual impairments have experienced improved quality of life through leisure engagement (Gwon et al., 2011; Nam, Cheong, & Choi, 2013). For example, Gwon and his colleagues emphasized the importance of physical activity as a means of improving quality of life as those study participants who were not involved in physical activities experienced higher levels of academic stress. In addition, Nam et al. (2013) provided evidence that participation in traditional music facilitated the experience of personal growth and resilience among Koreans who have visual impairments.

## Methods

This study adopted in-depth interviews as they allowed researchers to understand and explore dynamics of human behaviors and social interaction with others (Rubin & Rubin, 2005). This method is beneficial for researchers to examine various angles of life experiences among individuals with a disability and an illness (Sandelowski & Jones, 1996). Based on the purpose of our study and merits of in-depth interviews, this method is the most appropriate to respond to our research inquires.

### Participants and data procedure

A total of 14 adolescents who have visual impairments participated in this study. Of the participants, eight were male and six were female. Their ages ranged from 15 to 18 yearsold. Twelve of the participants were legally blind, while two had severe vision impairments and could not see words and letters in ordinary newsprint (see Table I). The participants voluntarily engaged in this study. For their participation in our study, we compensated each of them with approximately $20 USD. Storms and Loosveldt (2004) suggested that monetary reward is necessary as it can reduce the exclusion of minority population including low-income families and individuals with disabilities. To protect the participants’ identities, only pseudonyms were used. This study incorporated the theoretical saturation which refers to the process of gathering data till the point where no new information emerges (Guest, Bunce, & Johnson, 2006). The University Institutional Review Board approved this study.

**Table I.** Demographic characteristics of participants.

Name	Gender	Age	A Level of Blindness
Song	Female	15	Severe
Lee-L	Male	17	Blind
Park	Male	17	Blind
Kim-P	Male	18	Blind
Han	Female	16	Blind
Sun	Female	15	Severe
Kim	Male	17	Blind
Lee	Female	16	Blind
Young	Female	17	Blind
Yang	Male	17	Blind
Won-F	Female	15	Blind
Kim-F	Male	18	Blind
Jung	Male	17	Blind
Su	Male	18	Blind

To locate potential participants, we contacted a special education public school in a metropolitan area in Korea. With collaboration of schools, we held meetings with the special education teachers and their students in order to discuss the purpose and procedure of the study and follow the school policy as it relates to research projects. In addition, we placed a flyer on the school bulletin board explaining the study. When a participant expressed an interest in participating in the study, we held an individual meeting with the person in order to provide detailed information about the study and obtain consent forms from the individual and his or her parents.

### Interview protocol

This study used semi-structured, in-depth interviews. Each interview lasted between 90 and 120 minutes and was conducted in a location convenient for each participant. We adopted the content-mapping and content-mining interview questions suggested by Legard, Keegan, and Ward (2003) to capture the health benefits through leisure engagement among the participants. Examples of content-mapping questions were, “Please tell me what you like to do when you have free time” and “With whom do you participate in any activities?” To amplify probes and clarification related to health benefits through leisure, content-mini questions were asked. Examples included “Why do you participate in the activities you mentioned?”, “What benefits do you experience when participating in these activities?”, and “What role, if any, has these activities had in helping you deal with stress?” With the participants’ permissions, all of the interviews were audio-recorded using an MP3 player and transcribed verbatim. The primary investigator generated the raw data. The interviews were conducted in Korean and two researchers who are bilingual and experienced in qualitative research methodology validated the data translations from Korean to English.

### Data analysis

This study was theoretically based on the method of interpretative phenomenological analysis (IPA). IPA is “concerned with the detailed examination of personal lived experience, the meaning of experience to participants and how participants make sense of that experience” (Smith, 2011, p. 9). IPA features a combination of psychological, interpretative, and idiographic components (Smith, 2011). Smith, Flowers, and Larkin (2009) proposed this analysis is most appropriate to make meaning of participants’ particular life experiences with purposive sampling as well as researchers’ own perception, beliefs, and experiences. In this study, each participant was characterized by similar physical conditions and we were able to utilize our knowledge in inductively analyzing data. Based on this analysis, we followed the six data analysis steps were used as suggested by Rafique and Hunt (2015): (a) reading and rereading, (b) taking initial notes, (c) generating emergent themes, (d) developing connections among the themes, (e) comparing themes to the next case, and (f) identifying and interpreting themes across cases. Once we generated each theme, we used the constant comparative method that allowed us to constantly compare it with others (Merriam, 1998).

1. Generating raw data and reading and rereading. Each raw data transcript was generated and read and reread. By reading each transcript, we created an overall picture of each participant’s experience.
2. Taking initial notes. We included initial notes in the margins of the transcripts during the first step. These initial notes served as examples of possible themes related to health benefits through leisure engagement.
3. Generating emergent themes. Based on the initial notes, we sought to create emergent themes related to the consistent patterns and connections apparent in the transcripts.
4. Developing connections among the themes and generating sub-themes. We clustered emergent themes and then created sub-themes with direct quotes and narrative descriptions.
5. comparing themes to the next case. We compared emergent themes and subthemes with the next case. This process was repeated for all the cases.
6. Identifying and interpreting themes. An identification of themes across transcripts was performed. We interpreted the core themes and subthemes with labels.

### Trustworthiness of the qualitative research

Three major strategies were employed in order to increase the rigor of the qualitative research data conducted during this study. The first strategy was the member-checking strategy suggested by Peterson, Pape, and Eaton (2007). After interpreting the data, we held meetings with each participant in order to share our data interpretation with him or her. Thirteen participants voluntarily participated in the member checking process and expressed their satisfaction with the data interpretation. The second strategy used was the back-translation as guided by Suh, Kagan, and Strumpf (2009). Two bilingual professors who were not part of the research team were invited to verify the accuracy of the translations from Korean to English. Finally, each investigator specialized in qualitative research methodology with demonstrated qualitative expertise, which improved the trustworthiness of the data.

## Results

According to the participants, their main leisure activities were adaptive sports, recreational activities, music, Taekwondo, and art. Based on their experiences and statements, two salient themes were captured as health benefits resulting from leisure engagement: (a) psychological benefits and (b) personal growth. These positive outcomes can serve as vehicles for promoting health and wellbeing among participants.

### Psychological benefits

This theme highlights the psychological wellbeing that resulted from leisure engagement among participants. All of the participants mentioned that they gained various psychological benefits, such as positive emotions, happiness, and self-expression, through leisure engagement.

#### Positive emotions

All of the participants reported perceiving positive emotions by exhibiting leisure interests and demonstrating leisure skills and techniques. In particular, building confidence through leisure engagement generated positive feelings and emotions in participants. They mentioned that they built confidence by advancing and demonstrating their leisure skills and techniques, which resulted in positive emotions and feelings. For example, Young (female, 17) who regularly participated in arts and crafts said she was passionate about knitting and pencil drawing. She also mentioned that she demonstrated better skill sets in arts and these activities helped her increase her positive emotions related to her art skills.

In a similar manner, Park (male, 17) mentioned that he exerted a significant effort to earn a black belt and successfully achieved his goal. He disclosed that he gained his confidence through Taekwondo activity and focused on his ability and strengths in his life. Similar to Park’s experience, Yang (male, 17) practiced blind soccer as a member of a blind soccer team. He built self-confidence and self-esteem by mastering his soccer skills and techniques and, as a result, he experienced positive feelings about himself.

#### Happiness

All of the participants experienced a sense of happiness while engaging in leisure and used similar expressions of their happiness, including “I am so happy with what I am practicing [Taekwondo],” “doing art makes me so happy,” and “practicing soccer as a team makes me happy and satisfied.” They contented themselves by participating in leisure activities and experiencing a sense of joyfulness and enjoyment that resulted in happiness.

Most of the participants mentioned that they had limited occasions to interact with others with and without a disability. However, they believed that leisure provided rich opportunities to socialize with others and form a sense of peer relationship. By interacting with others, they perceived a sense of happiness. For example, Sun (female, 15) said, “I made lots of good friends here [art studio] and it made me happy.” She also mentioned that she developed and maintained friendships even after the program. Similarly, Song (female, 15) volunteered to offer violin lessons to others who had visual impairments, sharing that she connected with other students via her violin lessons and interacting with them made her feel rewarded and fulfilled.

#### Self-expression

Some participants mentioned that, in the past, they had experienced challenges communicating with others in regard to their feelings and emotions to release their stress and express their psychological concerns. Some of the participants disclosed that they acquired self-expression skills through leisure. In particular, participants who pursued music and art revealed that they developed self-expression skills. They articulated examples of improved self-expression skills through leisure, such as resolving conflicts, reducing stress, and developing social skills. For example, Lee-L (male, 17) stated

I enjoyed drawing and it helped me define what I was passionate about. I loved sharing my artwork with parents and teachers and believed I had a better relationship with my parents through my artwork.

He also mentioned that he was eager to express his feelings and emotions through his artwork.

Song (female, 15), who played the violin, stated that music helped her reduce her feelings of frustration, depression, and loneliness. She also mentioned that playing the violin and singing were means through which she was able to share her strength and music experience with other participants. In addition, she was involved with community-based music classes that helped her advance her music skills and interact with others.

In a similar manner, Won-F (female, 15) mentioned that she used music as a coping mechanism to deal with her stress. She said that by listening to music and learning how to play the piano, she reevaluated her feelings and emotions and refocused on her life objectives. As a result, she expressed her feelings more positively when she engaged in a conversation with others.

Based on the participants’ experiences and statements, they gained psychological benefits, such as positive emotions, happiness, and self-expression skills, through leisure engagement. The participants indicated that leisure provided them rich opportunities to establish friendships and develop coping strategies. In addition, the participants acquired advanced leisure skills and techniques and had aspirations to demonstrate them, which contributed to their self-esteem and confidence. Such positive experiences can lead to psychological wellbeing among participants.

### Personal growth

This theme describes the experience of personal growth through leisure engagement. The participants identified several challenges when they participated in leisure activities, such as limited resources and opportunities, adaptation challenges, and mobility issues. In spite of these challenges, they appreciated the opportunities to engage in leisure activities and used these challenges to develop a sense of perseverance. It seems that the participants explored their strengths and values through their participation in leisure and reevaluated their personal lives in new light.

#### Appreciation of life

All of the participants identified major challenges experienced in regard to their leisure engagement, including adaptation to physical settings, activity adaptations, dependence on others, and mobility issues. In spite of these challenges, they appreciated the opportunities to participate in leisure. For example, Kim (male, 17) said that he appreciated the opportunities he was provided to play with other children in adapted soccer and to participate in art and gardening. He mentioned that participation in such activities was meaningful and rewarding to him.

According to Song (female, 15)

Before I learned how to play the violin, I did not know what to do when I had free time … when I wake up in the morning, I touched my violin and say “thank you” because this violin means a lot to me. I appreciate the musical instrument as it [has] changed my life positively.

She also indicated that her appreciation of music helped to improve confidence and positive self-image.

Some of the participants mentioned that they appreciated the opportunities provided to them to demonstrate their leisure skills and techniques. For example, Park (male, 17) who achieved a black belt in Taekwondo said that he appreciated the opportunity to demonstrate his Taekwondo skills and techniques to advance to the next level. In addition, he stated that he appreciated his family’s support, as they demonstrated their support for Taekwondo events and festivals.

Similar to Park’s experience, Lee-L (male, 17) came to better appreciate his participation in adaptive sports because his parents were proud of his demonstration of his adaptive sports skills. He mentioned that, regardless of the outcomes of the games, he valued his participation of itself in adaptive sports and appreciated his family’s support of his engagement in them.

#### A sense of perseverance

All of the participants experienced obstacles and adversities due to being visually impaired in a context of leisure and mentioned that they were determined to develop the ability to persevere through challenges in order to enhance their mental toughness. For example, Kim (male, 17) said, “I sought for success constantly for my soccer performance.” He also mentioned that he persevered in his adaptive sports regardless of whether the circumstances were positive or negative. It appears as though the participants experienced mental toughness and mental preparation due to these challenges, which lead them to gain a sense of perseverance and self-efficacy.

Some of the participants mentioned that they exerted significant efforts to persistently succeed in their engagement in leisure. To this end, they showed self-determination and dedication to their activities. Such efforts helped them to develop a sense of perseverance. For example, Lee (female, 16) said,

I was determined and dedicated to engaging in art and music even though there were obstacles and challenges to doing that.

Similarly, Kim (male, 17) mentioned that he would not give up being a master in Taekwondo. He also believed that he developed coping strategies due to the challenges that he faced.

Lee-L (male, 17), who was a member of an adaptive soccer team, said that he experienced physical injuries when he competed with other teams and had the fear of being injured. In spite of this fear and his actual physical injuries, he continued to play and experienced satisfaction with his games. He also received emotional and social support from his teammates and family members.

These life examples showed that participants exhibited an appreciation of life through leisure engagement and sought to overcome any challenges associated with their visual impairments. It seems leisure serves as a catalyst for mental toughness and perseverance among participants.

## Discussion

This study was an initial exploration of health benefits as a result of leisure engagement among Korean adolescents who have visual impairments. The findings of this study indicated that Korean adolescents who have visual impairments gained psychological benefits and experienced personal growth through leisure engagement. This study indicated that leisure provides a context in which these individuals were able to demonstrate their leisure skills and techniques, improve their self-confidence, develop perseverance, and gain appreciation of their lives. In addition, it suggests that individuals with visual impairment can identify their strengths and interests and develop resilience to challenges and obstacles through leisure engagement.

Leisure scholars have provided evidence that leisure engagement promotes psychological wellbeing and life satisfaction among individuals with disabilities (Chun & Lee, 2010; Kim et al., 2015). The current study indicates that participants gain positive feelings, develop self-expression skills, and enhance their sense of happiness through leisure. Such positive outcome gained through leisure can contribute to life satisfaction. Furthermore, the findings of this study suggest that by improving confidence and self-esteem, individuals who have visual impairments can embrace opportunities for inclusion.

Previous studies have shown that individuals who have visual impairments use leisure as a way to cope with and adapt to psychological distress and stress (Huang, Chien, & Chung, 2013; Lee & Kim, 2013). The current study is aligned with these previous findings that various types of leisure helped participants develop the ability to cope with psychological distress and stress and, as a result, contribute to positive emotions and happiness. It appears that leisure provides rich opportunities for individuals who have visual impairments to socialize with others and improve self-esteem and confidence, which serve as a buffer against stress.

In a recent study by Park et al. (2015), individuals who have visual impairments used music and art as a means of self-expression and social interaction. In particular, they suggested that group music programs can help individuals who have visual impairments expand their interpersonal relationships and develop psycho-social coping strategies. The findings of the present study expand the body of knowledge that various leisure activities can be used as important therapeutic programs so that individuals with visual impairments can obtain skills to express their feelings positively and connect with other participants.

Several researchers (e.g., Chun & Lee, 2008; Iwasaki, Mactavish, & MacKay, 2005; Kleiber, Hutchinson, & Williams, 2002) have shown that leisure has facilitated personal growth in the midst of traumatic and stressful life events by providing individuals with disabilities or illnesses opportunities for positive engagements as well as to appreciate the value of life and develop interpersonal relationships. The findings of this study expanded the body of literature to show that participants appreciated their opportunities for leisure engagement and sought to overcome any challenges or obstacles to their leisure pursuits. As such, these experiences may lead to personal growth for the participants.

According to Tedeschi and Calhoun (1996), an appreciation of life is one of the important elements to facilitate stress-related growth among individuals who experience negative and traumatic life events. Chun and Lee (2008) suggested that leisure facilitated personal growth experiences among individuals with traumatic brain injuries, suggesting that participants expressed an appreciation for life through leisure. This study supports their findings by showing that adolescents who have visual impairments can experience positive changes following stressful life events.

### Limitations and future studies

Some limitations of this study need to be addressed. First, this study focused on Korean adolescents who have visual impairments, and multiple factors exist that influence their leisure participation, such as age, educational background, and gender. It may be interesting if future researchers investigate what factors influence leisure behaviors among individuals who have visual impairments.

The second limitation is related to the nature of the methodology used in this study. This study was designed to explore the health benefits gained through leisure engagement by Korean adolescents who have visual impairments, not generalize its findings. It would be beneficial for future researchers to quantitatively investigate the relationship between leisure and health benefits among Korean adolescents who have visual impairments.

Finally, this study did not differentiate types of visual impairment among individuals with visual impairments. There may be a difference in leisure tendencies between those born blind and those who lost their sight later in life. Future studies are needed to investigate the relationships among types of visual impairment, health and leisure.

### Practical implications and conclusion

Individuals who have visual impairments may experience barriers and challenges to participating in leisure, such as adaptation challenges, limited leisure resources, and environmental/structural barriers. Recreational therapists working with clients with visual impairments need to provide necessary adapted assistance to enable those who are visually impaired to fully engage in various leisure activities.

There are a variety of club activities and adapted sports organizations for individuals who have visual impairments. Recreational therapists need to encourage clients who aspire to advance their leisure skills and techniques to be part of adapted sports teams and clubs. They can build confidence and independence and create social bonding with other participants through adapted sports organizations.

Overall, this study has advanced the literature on leisure by suggesting that leisure can promote psychological wellbeing and health among Korean adolescents who have visual impairments. The findings suggest that leisure provides an avenue for the development of self-expression, leisure skills, perseverance, and a positive outlook that facilitate psychological benefits and personal growth. They also suggest that leisure can serve as a vehicle for promoting health and life satisfaction among Korean adolescents who have visual impairments.
